# Auditory Feedback Control of Vocal Pitch during Sustained Vocalization: A Cross-Sectional Study of Adult Aging

**DOI:** 10.1371/journal.pone.0022791

**Published:** 2011-07-25

**Authors:** Peng Liu, Zhaocong Chen, Jeffery A. Jones, Dongfeng Huang, Hanjun Liu

**Affiliations:** 1 Department of Rehabilitation Medicine, The First Affiliated Hospital, Sun Yat-sen University, Guangzhou, China; 2 Department of Psychology and Laurier Centre for Cognitive Neuroscience, Wilfrid Laurier University, Waterloo, Ontario, Canada; The University of Western Ontario, Canada

## Abstract

**Background:**

Auditory feedback has been demonstrated to play an important role in the control of voice fundamental frequency (F_0_), but the mechanisms underlying the processing of auditory feedback remain poorly understood. It has been well documented that young adults can use auditory feedback to stabilize their voice F_0_ by making compensatory responses to perturbations they hear in their vocal pitch feedback. However, little is known about the effects of aging on the processing of audio-vocal feedback during vocalization.

**Methodology/Principal Findings:**

In the present study, we recruited adults who were between 19 and 75 years of age and divided them into five age groups. Using a pitch-shift paradigm, the pitch of their vocal feedback was unexpectedly shifted ±50 or ±100 cents during sustained vocalization of the vowel sound/u/. Compensatory vocal F_0_ response magnitudes and latencies to pitch feedback perturbations were examined. A significant effect of age was found such that response magnitudes increased with increasing age until maximal values were reached for adults 51–60 years of age and then decreased for adults 61–75 years of age. Adults 51–60 years of age were also more sensitive to the direction and magnitude of the pitch feedback perturbations compared to younger adults.

**Conclusion:**

These findings demonstrate that the pitch-shift reflex systematically changes across the adult lifespan. Understanding aging-related changes to the role of auditory feedback is critically important for our theoretical understanding of speech production and the clinical applications of that knowledge.

## Introduction

During vocal communication, the fundamental frequency (F_0_) of one's voice is used to convey a large range of social information such as the emotional state of the speaker, whether an utterance is a statement or a question, and whether the speaker is being sarcastic or emphatic. Fine-tuned control of voice F_0_ is central to the skill of singing, and for tonal languages voice F_0_ is used to derive lexical or grammatical meaning. Although voice F_0_ represents a fundamental parameter for speech communication, the neural mechanisms underlying its control remain unclear. Auditory feedback (hearing your own voice during speech) is believed to play a critical role in F_0_ control by providing important information for the implementation of speech motor goals during speech articulation and for correcting for errors that occur during speech development and throughout life [1]–[3]. Previous research has demonstrated that when auditory feedback is missing, masked or altered during vocalization, the accuracy of voice F_0_ control is diminished [4]–[6].

A number of researchers have explored the role of auditory feedback in voice F_0_ control by exposing speakers to altered versions of their feedback [1], [7], [8]–[11]. During these experiments, the participants were asked to vocalize a vowel sound or a speech syllable while they heard their voice pitch unexpectedly altered in an upward or downward direction. These studies have consistently shown that speakers compensate for changes in voice pitch feedback: they lower their voice pitch when their feedback is shifted upward and increase their voice pitch when their feedback is shifted downward. It has been suggested that this response is reflexive because subjects seem to be unaware that they are changing their voice F_0_ so rapidly (∼100 ms) [8]; therefore, it is termed the pitch-shift reflex. This direction-specific vocal response to a pitch-shifted stimulus indicates that the audio-vocal system not only detects the direction of the pitch perturbation in auditory feedback, but also adjusts the response accordingly. Furthermore, multiple lines of evidence suggest that the audio-vocal system modulates vocal responses to pitch perturbations in auditory feedback according to the specific demands of the vocal task. For example, the magnitudes of vocal F_0_ responses are larger when people are singing a phrase compared to speaking a phrase [11]. The responses are also larger when speakers are producing a speech syllable compared to a vowel sound [12], and when they are vocalizing at a higher voice F_0_ level compared to a lower F_0_ level [9].

Despite the growing literature demonstrating the importance of auditory feedback and the role it plays in voice F_0_ control, very few studies have been conducted on how voice F_0_ control varies across different populations of people. Specifically, little work has addressed the effect of aging, despite the fact that the elderly population is the fastest growing segment, with people over the age of 65 years making up over 10% of the population in most countries [13]. Numerous studies have evaluated the effect of aging on speech production and have identified the acoustic changes associated with the aging voice [14], [15]–[17]. For example, age-related changes of the average voice F_0_ have been demonstrated for both men and women [14], [18], [19]. It has also been well documented that, during vowel phonation, people over 60 years of age exhibit significantly greater instability in the average voice F_0_ as reflected by higher standard deviations (SDs) than younger adults [16], [20], suggesting that elderly adults have a more variable voice F_0_ than young adults [13]. Aging-related changes in laryngeal muscles have also been found that include a loss of muscle mass, degeneration and decrease in fiber diameter [13], reduced laryngeal electromyography (EMG) activity, and decreased firing rates for the thyroarytenoid muscle [21]. In addition, people over 55 years of age differ from younger individuals in their neural representations of pure tones and speech sounds at the cortical level [22], [23]. For example, in response to speech sound stimuli, young adults (22–25 years) produced larger P1-N1 peak-to-peak amplitudes over the left temporal lobe relative to the right temporal lobe, while elderly adults (ages>55 years) produced symmetrical responses. However, since most of the previous pitch-shift studies were conducted with healthy young adults between 18–30 years of age, whether aging affects the processing of auditory feedback to control voice F_0_ remains unclear.

Recently, two pitch-shift studies showed that, as compared to young (18–30 years) and elderly adults (60–73 years), school children (7–12 years) produced significantly longer latencies of vocal F_0_ responses during sustained vocalization [24], [25]. In addition, the elderly participants produced significantly larger response magnitudes than school children and young adults [24]. These findings provide evidence that age is an important factor that contributes to the control of voice F_0_. However, since neither of these two studies included participants between the ages of 31 to 60 years, there are big gaps in our understanding of how the pitch-shift reflex changes over the adult lifespan. Clarifying the influence of age on the auditory feedback control of voice F_0_ is needed for a complete understanding of speech production as a whole. Moreover, increasing our understanding of how normal aging affects voice F_0_ control is clinically important and will have implications for the evaluation and treatment of many voice disorders associated with advancing age.

The present study was designed to investigate the aging-related changes in the auditory feedback control of voice F_0_ during sustained vocalization by answering the following questions. First, how do the vocal F_0_ responses vary over the adult lifespan? That is, do the response magnitudes steadily increase with advancing age, or is there a turning point at which they reach the minimal or maximal value? Second, at what age will people produce significantly different vocal F_0_ responses from those produced by young adults? To answer these questions, we recruited adult speakers between 19 and 75 years of age and divided them into five age groups. Using a pitch-shift paradigm similar to that used in previous studies [7], [25], the participants' vocal pitch feedback was unexpectedly shifted upward or downward with a magnitude of a half semi-tone or a full semi-tone. The magnitude of the pitch-feedback perturbation was manipulated in the present study because previous research has demonstrated a differential effect of pitch perturbation magnitude on the pitch-shift reflex across vocal tasks and participant populations [12], [25], [26]. By comparing the magnitudes and latencies of the pitch-shift reflex produced by the five age groups, the present cross-sectional study revealed how the aging process affects auditory feedback control of voice F_0_.

## Methods

### Ethics Statement

Informed consent forms were obtained from all subjects, and the research was approved by the Institutional Review Board of The First Affiliated Hospital at Sun Yat-sen University of China.

### Subjects

Sixty subjects (age: 19–75 years; 35 female and 25 male) participated in this experiment. They were divided into 5 age groups, with 12 subjects in each group: 19–30 years old (age: 19–25, mean age: 22 years, 5 female and 7 male), 31–40 years old (age: 31–40 years, mean age: 36 years, 6 female and 6 male), 41–50 years old (age: 41–50, mean age: 44 years, 7 female and 5 male), 51–60 years old (age: 51–59 years, mean age: 56 years, 9 female and 3 male), and 61–75 years old (age: 61–75 years, mean age: 69 years, 8 female and 4 male). Forty-two of the 60 subjects spoke Mandarin only and were incapable of speaking or understanding Cantonese. Eighteen subjects spoke both Cantonese and Mandarin, but Mandarin was the language they spoke in daily life. Of the 60 subjects, 48 passed the hearing screening test for pure tone frequencies of 500, 1000, 2000 and 4000 Hz at 25 dB hearing level (HL), and 12 passed the screening at the threshold of 40 dB HL. Of the subjects who failed the hearing screening test at 25 dB HL, 7 were from the 61–75 years group, 3 were from the 50–60 years group, 1 was from the 41–50 years group, and 1 was from the 31–40 years group. None of the participants reported a history of any speech, language or neurological disorders.

### Apparatus

Subjects were tested throughout the experiment in a sound-attenuated chamber. Their voice signals were transduced through a Genuine Shupu microphone (model SM-306), amplified with a MOTU Ultralite Mk3 firewire audio interface, pitch-shifted with an Eventide Eclipse Harmonizer, and then played back to subjects through headphones (model T20RP mkΠ). Prior to the testing, acoustic calibration was performed on the recording system to insure that vocal feedback was heard by subjects with a gain of 10 dB (sound pressure level, SPL) relative to the intensity of their true voice output. A Macintosh computer ran a custom-developed MIDI software program (Max/MSP, v.5.0 by Cycling 74) that triggered the Harmonizer to randomly pitch-shift the voice feedback upwards or downwards. The program also produced a transistor-transistor logical (TTL) control pulse to mark the onset and offset of pitch shifts. The vocal output, feedback and TTL control pulses were digitized at 10 kHz by a PowerLab A/D converter (model ML880, AD Instruments, Castle Hill, Australia), and recorded using LabChart software (v.7.0 by AD Instruments) on a second Macintosh computer.

### Procedure

**Figure 1:**
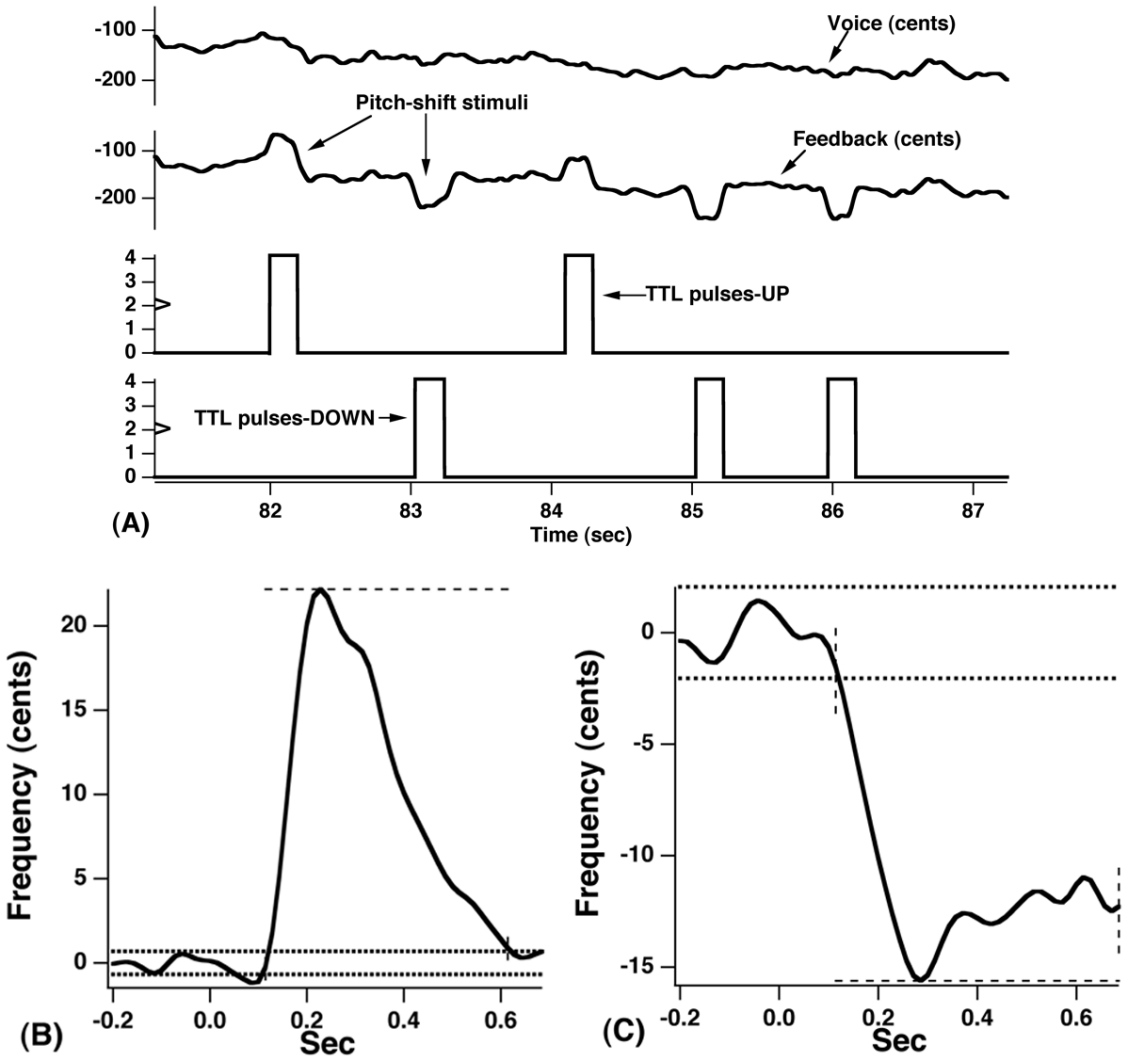
Contours of voice F_0_ (top trace), auditory feedback (middle trace), and TTL pulses (bottom traces) (A) and two representative averaged vocal responses to −100 cents (B) and +100 cents (C) pitch-shifted stimuli. For Figure 1A, the arrows in the contour of auditory feedback denote the upward and downward pitch-shifted stimuli (100 cents). For Figures 1B and 1C, horizontal dense dotted lines represent ±2 standard deviations of the pre-stimulus mean averaged F_0_. Vertical dashed lines indicate the onset and offset times of the responses. Horizontal sparse dashed lines indicate the response magnitude as reflected by the maximal or the minimal value of the average response. Time 0 represents the onset of pitch-shift perturbation.

The participants were instructed to vocalize/u/for approximately 5–6 seconds at their comfortable F_0_ level. During each vocalization, the participants' voice feedback was randomly pitch-shifted either upward or downward 5 times and instantaneously fed back to them through headphones (see Figure 1A). During each trial, the first pitch-shifted stimulus was presented with a delay of 500–1000 ms after vocal onset, and the succeeding stimuli occurred with an inter-stimulus interval of 700–900 ms. The sequencing of upward and downward stimuli was randomized within each block of trials. Each block consisted of 12 consecutive vocalizations, resulting in a total of 60 trials comprised of 30 upward and 30 downward pitch-shifted stimuli. During each block, the stimulus duration was fixed at 200 ms and the magnitude was held constant at 50 or 100 cents (100 cents = 1 semitone). The scale of pitch shifts in cent is logarithmically related to F_0_ (see below) and is constant relative to the absolute pitch produced by the subject. Pitch shifts of 50 and 100 cents were selected for this study because vocal responses produced by Mandarin speakers in response to these stimulus magnitudes were not significantly different from those produced by Cantonese speakers in a previous study [26]. Therefore, by selecting these pitch-shift magnitudes, the effects of any language differences across the subjects are minimized.

### Data analysis

During the offline analysis, the voice signal was first processed using Praat [27] to produce a train of pulses corresponding to the fundamental period of the voice waveform, and then transformed to an F_0_ contour waveform in a custom-developed IGOR PRO (v.6.0 by Wavemetrics, Inc., Lake Oswego, OR) software program. This F_0_ contour waveform was then converted from Hertz to a cent waveform using the following formula:



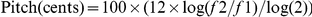
where 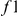 is an arbitrary reference note at 195.997 Hz (G4), and 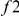 is the voice F_0_ in Hertz.

The voice F_0_ waveforms of all the trials within each block were time-aligned with the onset of the pitch-shifted stimulus (i.e. TTL pulse). They were sorted according to stimulus direction and averaged to generate one event-related response for each experimental condition per subject. For each average, a window with a pre-stimulus period of 200 ms (baseline F_0_) and a post-stimulus period of 700 ms was used. Prior to the averaging, each individual trial was waterfall displayed and visually inspected. Based on this visual inspection, trials with an unusually large amplitude, which could result from either signal processing errors or vocal interruption, were removed from further analysis. A valid response was defined as a change in the F_0_ contour that exceeded a value of two SDs of the pre-stimulus mean beginning no earlier than 60 ms after the stimulus onset and lasting at least 50 ms. Latency of the averaged response was measured as the time from the stimulus onset to the time at which the response exceeded 2 SDs above or below the pre-stimulus mean. Response magnitude was measured as the difference between the pre-stimulus mean and the highest or lowest value of the F_0_ contour following the response onset. A non-response was identified as a change in the F_0_ contour not meeting the criteria outlined above. This procedure for determining a valid vocal response is the same as that used in previous pitch-shift studies [10], [24]. Figures 1B and 1C show two representative averaged vocal responses to downward and upward pitch-shift stimuli. Note that for statistical analysis, response magnitudes to both upward and downward stimuli were recorded in terms of absolute magnitude, and are hereafter referred to as magnitude.

Significance tests of absolute values of response magnitude and latency were performed using SPSS (v. 16.0). Prior to the statistical analysis, tests of normality and homogeneity of variance were performed on the response magnitudes and latencies to verify that the assumptions of analysis of variance (ANOVA) were satisfied. A repeated-measures ANOVA (RM-ANOVA) was used for testing significant differences in response magnitude and latency across all conditions. If the assumption of sphericity was violated, probability values were corrected for multiple degrees of freedom using Greenhouse-Geisser and corrected *p* values were reported along with original degrees of freedom.

## Results

**Figure 2:**
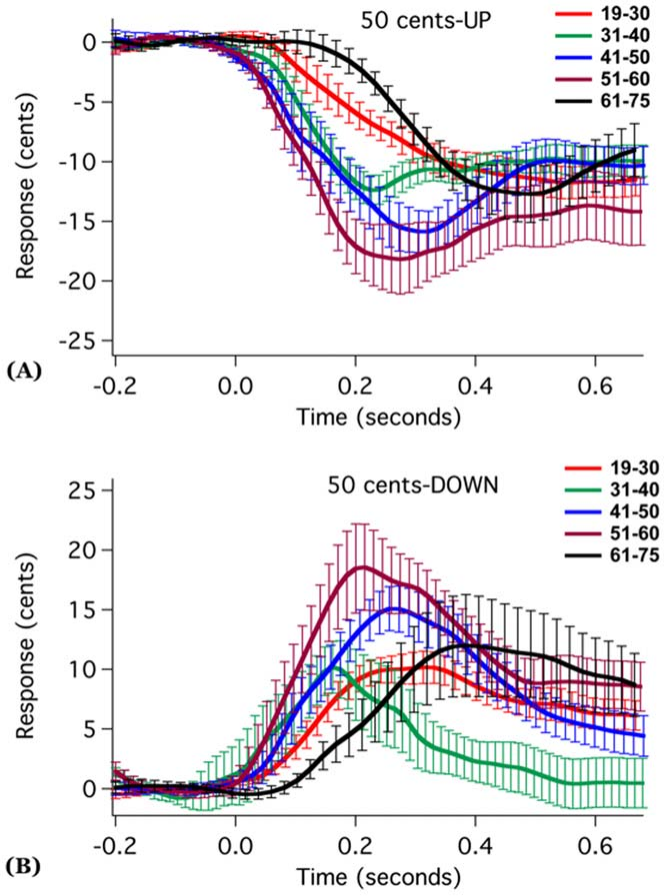
Grand averaged vocal responses over all subjects to upward (A) and downward (B) 50 cents pitch-shifted stimuli as a function of age. Solid lines represent average F_0_ contour and vertical bars represent the standard errors of averaged traces.

**Figure 3:**
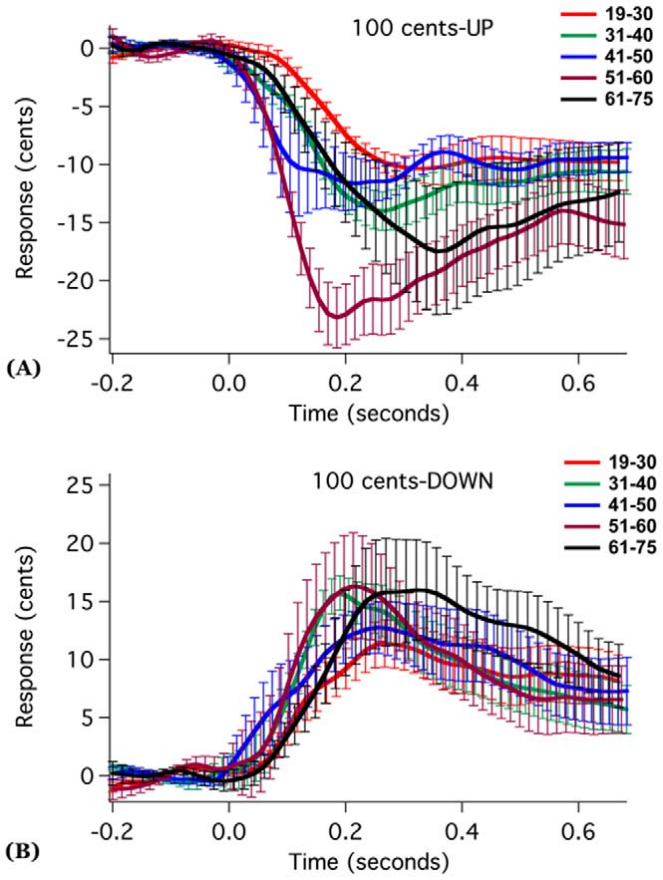
Grand averaged vocal responses over all subjects to upward (A) and downward (B) 100 cents pitch-shifted stimuli as a function of age. Solid lines represent average F_0_ contour and vertical bars represent the standard errors of averaged traces.

**Figure 4:**
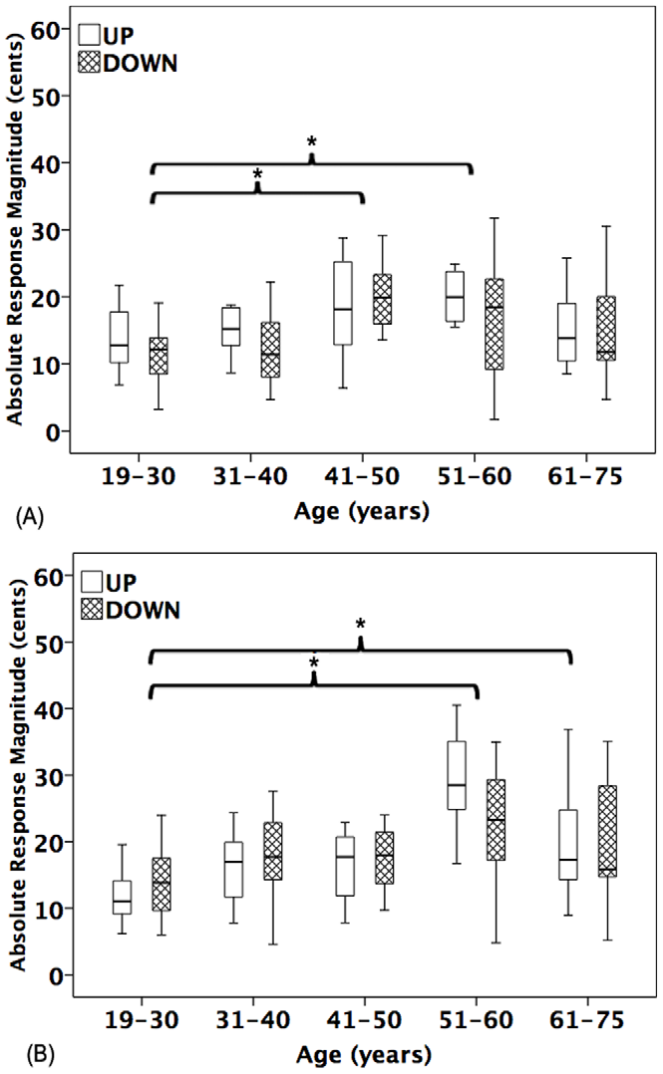
Boxplots of averaged vocal response magnitudes to 50 cents (A) and 100 cents (B) stimuli as a function of age and stimulus direction. The open and shaded boxplots denote the averaged absolute response magnitudes for upward and downward cents pitch-shifted stimuli, respectively. The asterisks indicate significant differences between conditions.

Figures 2 and 3 show the grand averaged voice F_0_ responses to 50 and 100 cents stimuli across age groups. As can be seen, the 51–60 year old group produced the largest response magnitudes, while the 19–30 year old group produced the smallest response magnitudes. In addition, for the 51–60 year old group, the upward 100 cents stimuli yielded larger response magnitudes than downward stimuli, but response magnitudes for the upward and downward directions for the 50 cents stimuli did not differ. The boxplots in Figure 4 showed the averaged response magnitude to 50 cents (A) and 100 cents stimuli (B) across age and stimulus direction. It can be seen that, for both the 50 and 100 cents stimuli, the response magnitudes increased with age until they reached a peak response magnitude for the 51–60 year old participants, and then decreased for people older than 60 years. Also as shown, as compared to young adults, the 51–60 and 61–75 year old groups showed higher variability in the response magnitude.

The averaged baseline F_0_ values and their SDs were measured from the baseline voice prior to the stimulus (i.e. 200 ms pre-stimulus period) across age groups. Although the 61–75 year old group produced the lowest voice baseline F_0_ values (194±61 Hz) compared to the other groups (19–30: 235±72 Hz; 31–40: 220±61 Hz; 41–50: 224±56 Hz; 51–60: 220±56 Hz), no significant differences were found across age groups (F(4, 55) = 0.702, p = 0.594). In addition, statistical analyses showed no systematic changes in baseline voice F_0_ SDs as a function of age (F(4, 55) = 2.268, p = 0.073). In order to determine if baseline voice F_0_ values should be entered as a covariate in the further analyses, regression analyses were performed to examine the correlation between response magnitude or latency and baseline voice F_0_. The results showed a significant negative correlation between response latency and baseline F_0_ value (t = −2.753, p = 0.006; r = −0.176), indicating that shorter response latencies were associated with higher baseline F_0_ values. However, no significant correlation was observed between response magnitude and baseline voice F_0_. Therefore, a repeated-measures analysis of covariance (RM-ANCOVA) with baseline voice F_0_ as a covariate was performed on the response latencies but not on the response magnitudes.

All data, including magnitude and latency, were logarithmically transformed prior to the statistical analyses to reduce any effects of variance heterogeneity. A three-way RM-ANOVA was performed on the response magnitude, and the results showed significant main effects of stimulus magnitude (F(1, 55) = 11.136, p = 0.002), age (F(4, 55)  = 6.882, p<0.001), as well as stimulus magnitude × age interaction (F(4, 55)  = 2.566, p = 0.048). No significant main effect was observed for stimulus direction (F(1, 55)  = 3.760, p = 0.058). A four-way RM-ANCOVA with baseline voice F_0_ as a covariate, stimulus magnitude and direction as within-subject variables, and age as a between-subject variable was performed. It revealed a significant main effect of baseline voice F_0_ on the response latency (F(1, 54)  = 8.049, p = 0.006). However, no significant main effects were observed for stimulus magnitude (F(1, 54)  = 0.131, p = 0.719), stimulus direction (F(1, 54)  = 0.126, p = 0.724), or age (F(4, 54)  = 0.956, p = 0.439). In addition, there were no significant interactions between baseline voice F0 and stimulus magnitude (F(1, 54)  = 0.029, p = 0.865), and stimulus direction (F(1, 54) = 0.079, p = 0.780).

Due to significant stimulus magnitude × age interactions for the response magnitude, separate two-way RM-ANOVAs were performed across each stimulus magnitude. For the 50 cents stimuli, one two-way (stimulus direction × age) RM-ANOVA showed a significant main effect of age on the response magnitude (F(4, 55)  = 3.932, p = 0.007), and post-hoc Bonferroni tests revealed that significantly smaller response magnitudes were produced by 19–30 year old group (12.5±4.6 cents) relative to the 41–50 year old (19.4±6.1 cents) (p = 0.007) and 51–60 year old groups (19.6±9.3 cents) (p = 0.048). A significant main effect of age on the response magnitude was also observed for the 100 cents stimuli (F(4, 55)  = 7.174, p<0.001), in which the 19–30 year old group (13.1±5.2 cents) produced significantly smaller response magnitudes than the 51–60 year old group (26.8±10.4 cents) (p<0.001) and the 61–75 year old group (20.3±10.3 cents) (p = 0.041). In addition, the 31–40 year old group (16.9±5.9 cents) produced significantly smaller response magnitudes than the 51–60 year old group (p = 0.015).

In addition, two-way (stimulus direction × stimulus magnitude) RM-ANOVAs were performed on the response magnitude across each age group. For the 51–60 year old group, significant main effects of stimulus magnitude were found (F(1, 11)  = 12.668, p = 0.004), indicating that the 100 cents stimuli (26.8±10.4 cents) yielded reliably larger response magnitudes than the 50 cents stimuli (19.6±9.3 cents). Upward stimuli (26.7±10.2 cents) produced significantly larger response magnitudes than downward stimuli (19.1±9.4 cents) (F(1, 11)  = 6.973, p = 0.023). Similarly, the 61–75 year old group produced significantly larger response magnitudes for the 100 cents stimuli (20.3±10.3 cents) than for the 50 cents stimuli (15.5±7.1 cents) (F(1, 11)  = 7.421, p = 0.020). For the other age groups, no significant main effects of stimulus magnitude or stimulus direction were found.

## Discussion

This cross-sectional study investigated the effects of aging on auditory feedback control of vocal pitch during sustained vocalization. The results showed a systematic change in the magnitude of vocal F_0_ responses as a function of age. The vocal F_0_ response magnitudes increased gradually with advancing age and reached the maximal value at 51–60 years of age and then decreased at 61–75 years of age. Moreover, the pattern of aging-related vocal F_0_ responses varied as a function of stimulus magnitude. As compared to 19–30 year old adults, significantly larger response magnitudes were produced by 41–50 and 51–60 year old adults for 50 cents stimuli, and by 51–60 and 61–75 year old adults for 100 cents stimuli. These findings reinforce the results of Liu et al. [24], [25] who reported the effect of age on vocal F_0_ responses from childhood to adulthood, and demonstrate that the pitch-shift reflex changes systematically as people age from young adulthood through to elderly adulthood.

We conducted the present experiment with two main questions in mind. First, how did the vocal F_0_ responses vary with advancing age? The answer to this question is as follows: from young adulthood to elderly adulthood, the magnitude of vocal F_0_ responses did not increase continuously; instead there was a turning point at which vocal F_0_ response reached a maximal value, and then began to decrease with advancing age. This turning point where vocal F_0_ response achieved the maximal magnitude was at 51–60 years of age. Interestingly, it was reported that the average F_0_ of speaking voice for men gradually drops until the fifth decade of life and then begins to increase as age increases [18], [19]. However, our statistical analyses showed that the changes in voice F_0_ did not contribute to the modulation of vocal response magnitudes across age groups. In the earlier study by Liu et al. [24], the age distributions of participants were not adequate enough to determine the aging-related changes in the pitch-shift reflex across the entire adult lifespan. This study is the first to report the turning point in life when the pattern of increasing vocal F_0_ responses to pitch-shift perturbations reverses. It has been shown that healthy people produce significantly different vocal responses than individuals with neurological disorders such as Parkinson's disease [28] and autism spectrum disorders [29]. These findings suggest that the vocal F_0_ response to pitch-shift perturbations may potentially serve as an indicator for the diagnosis of voice disorders. If this potential is realized, it will be important to take the turning point in the aging-related vocal responses that we have identified into account when developing these types of diagnostic tools.

Our second question was as follows: at what age would the vocal F_0_ responses of aging adults significantly differ from the responses observed for adults in our youngest age range? The answer is that it depends on the magnitude of the pitch-shift perturbation. As compared to young adults (19–30 years), the 41–50 and 51–60 year old groups had significantly larger response magnitudes when they heard their auditory feedback shifted 50 cents. On the other hand, the 51–60 and 61–75 year old groups had significantly larger responses than the youngest group when they heard their feedback shifted 100 cents. Thus, response magnitudes for the smaller (50 cents) shifts increased until the 41–50 years of age range, plateaued and then decreased in people between 61–75 years of age. However, response magnitudes to the larger 100 cents shifts increased until 51–60 years and then finally decreased in 61–75 year olds, but were still significantly different than the smaller responses made by young adults. This finding is consistent with the results of a previous study, which showed that a 60–73 year old group produced significantly larger vocal responses to pitch perturbations than a 19–21 year old group [24].

Aging-related changes in speech production and perception may be the result of a combination of age-related changes that occur in neurological, respiratory, articulatory and muscular systems [13]. Therefore, at this time it is difficult to identify the specific cause of the larger vocal responses produced by the elderly adults. However, one possible reason may be the aging-related physical changes that occur specifically in the vocal folds. A recent study that the shows that the cricothyroid (CT) and thyroarytenoid (TA) muscles, which control voice F_0_ by regulating the length, tension and three-dimensional geometry of the vocal folds, are involved in generating the compensatory vocal responses to pitch-shifted voice feedback [30]. With advancing age, the larynx possesses greater mass and larger internal stiffness of the vocal folds [20], which may decrease the accuracy of vocal motor control [31]. This decreased accuracy may have led to the larger magnitude and more variable vocal F_0_ responses observed in the present study. One may argue that the degradation of muscle composition and innervation in elderly adults may weaken some laryngeal muscles such as CT or TA, which should result in smaller rather than larger vocal responses compared with young adults. But the interaction between laryngeal muscles and voice F_0_ control may be not that simple as expected. It was reported that, although a decrease or increase in CT and TA muscle activity corresponded to the direction of voice F_0_ response during falsetto vocalization, this relationship was not observed at the conversational level, indicating that even the same laryngeal muscle may function differently in the modulation of vocal responses across tasks [30]. Therefore, how physiological changes in individual laryngeal muscles affect the pitch shift reflex is still unclear and needs to be explored in further experiments.

Another possible explanation for the larger responses in elderly adults is that an increase in age is associated with a decrease in the capacity of cortical and subcortical systems to inhibit responses to repetitive auditory stimuli [32]. It has been shown that there is a substantial reduction of gray matter volume with age [33], which may be due to a decrease in synaptic arbors and in the number of synapses [34]. The decreased number of inhibitory synapses in the aged brain could lead to larger response to repetitive stimulations, as found in an aging-related auditory perception study that demonstrated that elderly adults produced larger magnetoencephalography (MEG) responses to pure tones [35]. These findings could possibly explain the larger vocal responses to pitch-feedback perturbations we observed in people over 51 years old as compared to the younger adults. Since the mechanisms underlying auditory feedback control of voice F_0_ remain unknown, further studies should be conducted to investigate how the aging process affects the pitch-shift reflex at the peripheral and central level.

In the previous pitch-shift studies that involved young adults, the modulation of vocal response magnitude as a function of stimulus magnitude or direction during sustained vocalization was rarely reported [10], [12], [25]. The present findings are complementary to those studies in that the magnitudes of vocal F_0_ responses produced by young adults were not systematically modulated as the pitch-shifted stimuli varied from 50 to 100 cents. However, as mentioned, an effect of stimulus magnitude was observed for the older adults who were between 51–60 and 61–75 years of age. In addition, the 51–60 year old group produced larger responses to upward stimuli as compared to downward stimuli. Thus, as people get older, they not only respond to pitch feedback perturbations with larger response magnitudes than do younger adults, but they also adjust their responses according to the physical properties of the stimuli. Such a systematic change in the reflexive compensation for pitch errors is not only affected by age-related changes in anatomy and physiology of the speech production system, but it also appears to be related to the way the aging brain processes errors of different sizes. Auditory feedback regarding voice F_0_ appears to be used to modify motor plans to accommodate the effects of normal aging on the cognitive and sensory processing necessary for speech production. Once the effects of aging are fully described, clinicians will need to be aware of these age-related changes so that the effects of normal aging are not confused with changes associated with disease processes.

It is noteworthy that, as can be seen in Figure 4, elderly adults showed larger variability of response magnitude compared to young adults. Similar results were also found in another study, where greater variability of vocal F_0_ responses was associated with elderly adults as compared to school children and young adults [24]. Given the previously reported greater instability of voice F_0_ in adults over 60 years of age [16], [20], it was speculated that the frequency of voice F_0_ or variability of voice F_0_ might contribute to the variability in the magnitude of the pitch-shift reflex across age groups. However, our results showed that the magnitude of pitch-shift reflex was not correlated with baseline voice F_0_ or its SD, suggesting that variability in the response magnitude may not result from the aging-related changes in voice F_0_. This may not be surprising since large inter-subject variability is found in vocal responses to pitch feedback perturbations even in young adults [7], [36], [37]. For example, Burnett et al. [7] found that a large range in the magnitude of vocal F_0_ responses (2.6 to 100.3 cents) in young adults (18–22 years of age). Since the mechanisms that are responsible for the inter-subject variability of the pitch-shift reflex are unclear, future experiments should be conducted to address this issue.

In addition, the present study showed no statistically significant age-related changes in the average voice F_0_ produced, or the variability of F_0_ during the baseline periods of the utterances. These results are comparable with the data reported in some previous research [38]. However, other research has demonstrated that elderly people over 60 years old exhibit significantly higher variability in their average voice F_0_ than younger adults [16], [20]. One possible reason for this inconsistency is the different ways variability has been measured across studies. For example, in the present study, the mean and SD of voice F_0_ produced during the 200-ms baseline voice prior to the stimulus were evaluated, and statistical analyses were performed on the pooled data from both men and women. By contrast, these values in other studies were measured from either an entire sustained vowel, phrase or passage [16], [38], and the factor of sex was evaluated during the statistical analyses. It should also be pointed out that unlike these previous studies, determining age-related differences in average F_0_ was not a primary research question for this study because exposure to multiple perturbations to voice pitch feedback might affect these measures. Thus, any conclusions regarding age-related changes in average voice F_0_ and its variability should be made cautiously.

With regard to response latency, no effect of aging was observed across the conditions. This finding parallels the results of a previous study [24], in which no significant differences were found in the response latency as a function of age in adults. It was reported, however, that school-age children produced longer response latencies than young and elderly adults [24], [25]. Collectively, these results imply that response latency may index the maturation of the audio-vocal function during childhood given the differences observed between children and adults, and the consistency of response latencies across all the adult age ranges examined in this study. It should be noted that some electrophysiological studies have demonstrated that the latency of neural responses to auditory stimuli increases with increasing age [38]–[40], and that this increase may be the consequence of the neuronal loss in the aging brain [41]. Although the aging-related slowing down was found in the cortical processing of auditory stimuli, behavioral reaction times of the elderly adults were not significantly different from those of young adults [38], indicating that the aging-related delay only occurred at the cortical level of auditory processing but not in the motor response itself. It was suggested that the increased latency of neural responses might be due to delayed processing in the auditory pathway rather than as a result of delays in overall cognitive processing [42]. Since the pitch-shift reflex is a motor response that corrects for pitch errors in auditory feedback during vocalization, it may be that later cognitive processing may compensate for delays in earlier auditory cortical processing, which may explain the absence of an aging effect on the latency of the behavioral vocal responses in the present study.

A primary limitation of the present study is that we did not include sex as a between-subject factor to test its contribution to the aging-related pitch regulation. It has been well documented that men differ from women in the speech acoustic changes that accompany aging. For example, men and women show differences in laryngeal lowering and vocal tract lengthening that occur due to aging and this leads to different changes in their vowel acoustics [43], [44]. As well, men and women adjust their speech differentially to accommodate the respiratory and laryngeal changes that occur as part of the aging process [43], [45]. One of our recent pitch-shift studies that involved young adults showed that vocal F_0_ responses varied as function of sex, with men producing larger vocal responses than women [46]. Thus, the interaction between sex and the aging process on with respect to the pitch-shift reflex should be further studied.

### Conclusion

This cross-sectional study investigated the audio-vocal feedback control of pitch across the adult lifespan during sustained vocalization. The present findings demonstrate a significant effect of aging on the vocal F_0_ response: 19–30 year old adults produced significantly smaller response magnitudes than adults over 41 years of age. Moreover, there appears to be a turning point at which aging-related changes modify the pitch-shift reflex: the magnitude of responses to pitch-shifted feedback continuously increased and reached maximal values for 51–60 year old adults and then significantly decreased for 61–75 year old adults. Overall, the results of this study indicate that aging affects the auditory feedback control of vocal pitch during sustained vocalization. Given the importance of feedback processing, these changes are sure to interact with the other significant changes that occur in the sensory and motor systems involved in speech production.
